# Field Survey of Freshwater Invertebrates Reveals That Several Groups Are Potential Carriers of the Fungal Pathogen *Batrachochytrium dendrobatidis*

**DOI:** 10.1002/ece3.73513

**Published:** 2026-04-27

**Authors:** Amanda Poh, Perran A. Ross, Venice Chan, Taegan McMahon, Caitlin L. Nordheim‐Maestas, Laura A. Brannelly

**Affiliations:** ^1^ Melbourne Veterinary School, Faculty of Science The University of Melbourne Werribee Victoria Australia; ^2^ School of BioSciences, Faculty of Science The University of Melbourne Parkville Victoria Australia; ^3^ Connecticut College New London Connecticut USA; ^4^ University of California, Santa Barbara Santa Barbara California USA

**Keywords:** chytridiomycosis, disease diagnostics, DNA extraction, invertebrate vectors, wildlife disease

## Abstract

The amphibian disease chytridiomycosis, caused by the fungal pathogen *Batrachochytrium dendrobatidis* (*Bd*), has devastated global amphibian biodiversity. The specific reasons for its rapid global spread, especially to pristine areas, are not well understood. Freshwater invertebrates might function as carriers of *Bd*, but the diversity and extent of invertebrate species that might vector *Bd* in the wild is unknown. Thus, broad surveys of freshwater invertebrates at known *Bd‐*positive sites are needed to understand the potential risk and scope of these potential pathogen carriers. In this study, we aimed to identify potential invertebrate carriers of *Bd* in Victoria, Australia. We collected a wide variety of freshwater invertebrates from sites with vulnerable *Litoria* frog populations, batch‐pooled the samples and screened them for *Bd* DNA presence via qPCR. We detected *Bd* DNA presence in 2.1% of the batch‐pooled samples, from the families Chironomidae (non‐biting midges), Hydrophilidae (water scavenger beetles) and Corixidae (water boatmen). The detection of *Bd* in field‐collected invertebrates across three orders indicates that *Bd* might be more taxonomically widespread than previously recognised. These invertebrate taxa are ideal candidates for future vector competence studies, which are necessary to understand the implications for amphibian conservation.

## Abstract 2

Amphibians are dying around the world due to the devastating disease chytridiomycosis, and the spread of this disease might be caused by non‐amphibians, like invertebrates. We found three new invertebrate taxa that are positive for the pathogen that causes chytridiomycosis, indicating that invertebrate carriers might be important in the spread of this disease.

## 1 Introduction

Chytridiomycosis is a deadly amphibian disease caused by the fungal pathogen *Batrachochytrium dendrobatidis* (*Bd*). *Bd* has caused mass amphibian die‐offs and global declines in over 500 amphibian species and at least 90 extinctions, making it the worst wildlife disease known to science in terms of its impacts on vertebrate biodiversity (Scheele et al. 2019). In Australia, almost 20% of native amphibian species declined or have gone extinct due to chytridiomycosis (Scheele et al. 2017).

Chytridiomycosis is now present on every continent except Antarctica, although distribution across continents is patchy and some disease‐free sites with high amphibian biodiversity exist (Fisher et al. 2009; Monzon et al. 2020). While the mechanisms for the spread of *Bd* across the globe are not fully understood (Fisher et al. 2009; Rosenblum et al. 2013), human movement and the commercial amphibian trade are believed to be the main factors behind the global spread of chytridiomycosis (Rohr et al. 2011), and many countries have since implemented biosecurity measures to regulate the movement of amphibians (Department of the Environment and Energy 2016; Fisher and Garner 2007; Kriger and Hero 2009).

Despite increased amphibian biosecurity trade regulations, the rate of *Bd* discovery in new species and locations has not slowed (Monzon et al. 2020), raising concerns that *Bd* might be spreading through non‐amphibian alternative hosts. *Bd* has also caused severe declines and extinctions during outbreaks in pristine and inaccessible forests that are unlikely to be caused by human movement and amphibian trade (Becker and Zamudio 2011). While *Bd* is widespread, there remain several disease‐free regions that are climatically suitable for the fungus, including parts of Madagascar (Bletz et al. 2015; Kolby 2014; Kolby et al. 2015), Papua New Guinea (Bower et al. 2019) and certain areas in Australia such as Cape York in Queensland and western Tasmania (Murray et al. 2010). Effective biosecurity is the only way to protect these few bastions of amphibian biodiversity from the arrival of *Bd*, which is expected to have catastrophic effects on naïve amphibian populations in these areas (Bower et al. 2019). Current biosecurity protocols do not consider the role of non‐amphibian vectors, leaving a potentially critical gap in the protection of disease‐free regions.

Several non‐amphibian vertebrates have been suggested as potential vectors of *Bd*. These include waterfowl (Burrowes and De la Riva 2017; Garmyn et al. 2012; Johnson and Speare 2005), reptiles (Kilburn et al. 2011) and invertebrates including crayfish (Brannelly, McMahon, et al. 2015b; McMahon et al. 2013), fruit flies (Byer et al. 2024), biting midges (Toledo et al. 2021), mosquitoes (Gould et al. 2019; Reinhold et al. 2023) and nematodes (Shapard et al. 2012). Most of these organisms have not been demonstrated to be competent vectors through both laboratory transmission trials and field prevalence data, with the exception of *Procambarus alleni* and *P. clarkii* crayfish species. *P. alleni* and *clarkii* have been found positive with *Bd* infection through field surveys, are susceptible to *Bd* infection in laboratory experiments and can transmit infection to frogs (Brannelly, McMahon, et al. 2015b; McMahon et al. 2013). While there are some examples of possible additional invertebrate hosts of *Bd*, there are no published accounts of invertebrates testing positive for *Bd* in Australia. Broad taxonomic surveys are a key first step in understanding the risk of invertebrate carriers of *Bd* in Australia. Once species or taxonomic groups have been identified as possible vectors of *Bd*, follow‐up studies can be conducted to identify if the species can maintain *Bd* for an extended period, and transmit the pathogen to other susceptible hosts, like amphibians.

In this study, we aimed to identify potential invertebrate carriers of *Bd* in Victoria, Australia. We did this by collecting a wide variety of freshwater invertebrates from *Bd* positive sites with known populations of vulnerable *Litoria* frogs during the breeding period when *Bd* infection in the frogs is known to be high. At these sites, we collected invertebrate samples as well as frog samples and tested for *Bd* DNA presence using DNA extraction and qPCR. While detection of *Bd* via quantitative PCR (qPCR) alone cannot confirm that invertebrates are carriers of *Bd*, this broad taxonomic survey is necessary to identify targets for future studies, given that very little is currently known about which invertebrates might act as pathogen carriers. Furthermore, having prevalence data in the field is critical to understanding pathogen and disease dynamics in susceptible hosts as well as transmission risk of the invertebrate carriers to susceptible hosts. The invertebrate taxa that were positive for *Bd* DNA can be tested for transmission risk in future studies. This research is an important first step in determining invertebrate carriers of *Bd* and could play an important role in the disease dynamics of susceptible hosts of this global pathogen.

## 2 Methods

### 2.1 Study Sites and Target Frog Species

We conducted nocturnal frog surveys where we collected amphibian skin swabs and simultaneously collected invertebrate samples from freshwater habitats at sites known to contain populations of vulnerable *Litoria* species: *L. aurea* (Green and Golden Bell Frogs) and *L. raniformis* (Growling Grass Frogs). Both species are listed as vulnerable in Victoria under the Environment, Protection and Biodiversity Conservation Act 1999 (EPBC Act).

We selected these two species of frogs to sample as they have experienced severe declines due to chytridiomycosis, and the data from the collection of amphibian skin swabs for this study will contribute to ongoing disease monitoring efforts (Heard et al. 2012; Osborne et al. 2014). The detection of *Bd* presence in amphibians living sympatric to the invertebrates at the survey sites allows us to compare pathogen prevalence between sampled frogs and invertebrates, providing context for the detection of *Bd* in invertebrate hosts that share habitat with declining and protected frog species. We present amphibian *Bd* prevalence at each sample location and timepoint to demonstrate that each site was positive for *Bd* infection at the time of the invertebrate surveys. Furthermore, by surveying at a known *Bd* positive site with declining frogs, we are specifically targeting sites with a high likelihood of *Bd* in invertebrates, should they be possible carriers of *Bd*.

Exact site locations are not stated for the protection of these listed species (Lindenmayer and Scheele 2017). Site 1 is a water treatment plant containing a population of *L. aurea* located in Gippsland, Victoria. Sampling for *L. aurea* frogs and invertebrates was conducted in and around the wastewater treatment lagoons and the fringing vegetation. Sampling was conducted over 4 nights in September 2024. Site 2 is an industrial quarry and landfill containing a population of *L. raniformis* in the northern suburbs of metropolitan Melbourne, Victoria. Site 3 is near a water treatment plant containing a population of *L. raniformis* located in the western suburbs of metropolitan Melbourne, Victoria. Sampling for *L. raniformis* frogs and invertebrates was conducted around several still freshwater pools and the fringing vegetation. Sampling at Sites 2 and 3 was conducted once a month from September 2024 to January 2025.

### 2.2 Frog Sampling

The field survey for wild frogs involved surveyors with spotlights searching for amphibians on the bank of the pond and wading in water bodies. Any frog of the target species ( *L. aurea* at Site 1, and *L. raniformis* at Sites 2 and 3) encountered was caught with a clean nitrile‐gloved hand and kept individually in a new plastic zip bag. Gloves were changed between frogs for hygiene and biosecurity reasons, to reduce pathogen spread (see below). Each frog was measured (weighted to the nearest 0.5 g using a spring scales; snout to vent length measured to the nearest 0.1 mm using dial callipers) and swabbed with a sterile rayon‐tipped swab (MW113, Medical Wire & Equipment, Wiltshire, England) following standardised skin swabbing protocol for disease diagnostics: 5 strokes on each of the frog dorsum, venter, sides, thighs, hands and feet, while rotating the swab (Brannelly, Wetzel, West, and Richards‐Zawacki 2020a). After processing, the frogs were released to the site of capture. Swabs were frozen at −20°C until used in DNA extraction.

### 2.3 Invertebrate Sampling

We sampled freshwater invertebrates in and near the ponds opportunistically for 1–2 h per sample collection time. The aim was to sample a wide variety of taxa present at the site; therefore, we walked around the edge of the pond (approximately 10 m transects along 2 edges of each pond) and sampled where it was safe and accessible to do so. We sampled freshwater macroinvertebrates along the edges of waterbodies (up to 0.5 m deep) by sweeping with an aquatic dipnet (EANET1, Australian Entomological Supplies, mesh size 0.9 × 0.3 mm). We disturbed the bottom sediment by dragging the flat edge of the aquatic net along the bottom, and then immediately swept the water above the disturbed sediment. Any freshwater macroinvertebrates caught in the net were sorted from sediment and rinsed with pond water and placed into separate new microcentrifuge tubes containing 100% ethanol, where they were preserved until DNA extraction. Visually similar invertebrates were grouped into the same tubes. Freshwater snails were not collected as they have already been studied and identified to be unlikely hosts of *Bd* (McMahon et al. 2021). As we walked the perimeter of the pond, we also collected flying insects resting in vegetation fringing waterbodies by rustling and sweeping over the vegetation with a butterfly net (EBNET2, Australian Entomological Supplies, mesh size 0.9 × 0.3 mm). These insects were sorted and preserved in separate new microcentrifuge tubes containing 100% ethanol until DNA extraction. Invertebrates that were large (> 2.5 cm) were kept in clean plastic zip bags and frozen at −20°C until DNA extraction. After we returned to the laboratory, we visually identified invertebrate samples to the lowest possible taxonomic grouping using an identification guide for freshwater macroinvertebrates in Australia (Gooderham and Tsyrlin 2002). Our goal was to assess at least 30 individuals per invertebrate group, and we sampled at most 30 individuals per invertebrate group per sample timepoint. Based on our samples collected across the survey and our sample size goals, invertebrates were grouped by taxonomic family.

### 2.4 Disinfection of Equipment

Contamination among individuals and sites is a very important concern for this type of study; therefore, the utmost caution was observed for frog surveys and invertebrate collections. After each survey, we disinfected our waders, boots, nets and other equipment with F10 SC Veterinary Disinfectant (3%), which is known to be an effective decontaminant against *Bd* (Webb et al. 2007; Murray et al. 2011). We rinsed the disinfected equipment twice with water and allowed it to dry completely before surveying a new site.

### 2.5 Disease Diagnostic Testing of Frog Skin Swab Samples

DNA was extracted from amphibian skin swabs using PrepMan Ultra (Thermo Fisher Scientific, Waltham, Massachusetts, United States) according to the manufacturer's protocol (with minor modifications as done by Brannelly, Wetzel, West, and Richards‐Zawacki 2020a). Briefly, we added 50 μL of PrepMan Ultra sample reagent with 30–40 mg of 0.5 mm silica beads to each sample, beat the samples at 30 Hz for 2 min using a cell homogeniser (TissueLyser II; QIAGEN, Hilden, Germany), incubated them at 95°C for 10 min, centrifuged them and collected the supernatant. DNA extracts were diluted 6:100 in molecular‐grade water prior to qPCR analysis. For each round of extraction, we also extracted a negative (a clean rayon‐tipped swab) and positive control (a swab dipped in *Bd* culture broth).

DNA extracts were analysed for the presence of *Bd* DNA in singlicate using qPCR following Boyle et al. (2004) with minor modifications. Using a Rotorgene Q (5‐Plex HRM; QIAGEN, Hilden, Germany), we used a 15 μL reaction volume, comprising 5 μL of diluted template DNA, 7.5 μL of 1× Sensi‐fast Lo‐ROX Mix, and final reaction concentrations of 900 nM PCR primers (ITS1‐3 and 5.8S Chytr), 250 nM Chytr MGB2 FAM‐labelled probe and 400 ng / μL BSA. Each qPCR reaction plate included a series of 7 plasmid‐based *Bd* standards (Pisces Molecular, 4.2, 42, 420, 4200, 42,000, 420,000 and 4,200,000 ITS DNA copies per reaction), and a no template control (pure PCR‐grade water). *Bd* load refers to the number of *Bd* ITS copies present in the entire extraction sample, as calculated from the ITS copies detected by qPCR and extrapolated to account for dilution of template DNA (e.g., multiplied by 166.67). All extraction positive controls were qPCR positive for *Bd* DNA which indicates that our extractions were successful. All extraction negative controls were negative for *Bd* DNA, indicating that our extraction did not have cross‐contamination. And our qPCR no template negative controls were negative for *Bd* DNA, indicating that our qPCR preparation did not have cross‐contamination.

### 2.6 DNA Extraction and qPCR of Invertebrates

The only larger invertebrates (> 2.5 cm) that we processed were dragonfly larvae. For these samples, we swabbed and extracted the DNA from the external swabs (similarly to how we processed amphibian skin swabs above). A singular swab was used to swab 3–5 dragonfly larvae, and each larva was swabbed following the same protocol. We used a single swab rather than batch‐pooling the swab samples because it makes extraction with Prepman Ultra easier (e.g., more rayon‐tipped swabs within the extraction soak up more of the extraction liquid). This involved thoroughly swabbing the dragonfly larva over their entire body, five strokes per body section, including dorsal and ventral surfaces, limbs, wing buds, head and mouthparts. When swabbing the mouthparts we extended the labium to gain full access to the labial palps, teeth, the entire length of the labium, labrum and mandibles.

Smaller invertebrates were grouped together based on the lowest taxonomic group they could be identified to (e.g., Family), life stage, and the site and timepoint of collection. For each extraction, we removed 3–10 invertebrates (depending on the total number available) from the ethanol sample tube, and patted the samples dry using a Kim wipe. These samples were placed in ethanol, which likely washed off the transient zoospores, and then the samples were patted dry before extraction. Furthermore, transient and environmental *Bd* is difficult to detect, and eDNA studies have shown that 500–1000 mL of water is required to be filtered to accurately detect the presence of zoospores in waterbodies (Brannelly, Wetzel, Ohmer, et al. 2020b). It is unlikely that positive samples detected in this study are due to transient or environmental *Bd*.

Once the invertebrates were patted dry to reduce the amount of ethanol in the extraction, we processed the samples by bead‐beating at 25 Hz for 4 min (TissueLyser II; QIAGEN, Hilden, Germany). We used two 3‐mm glass beads for small invertebrates (< 1.5 cm body size, including amphipods, water boatmen, etc.), and added an additional 6 mm glass bead for larger invertebrates (1.5–2.5 cm body size) or invertebrates that were not well homogenised after bead‐beating with 3‐mm glass beads. We added 0–200 μL phosphate buffer saline (PBS), depending on the volume of invertebrates added, such that a full 10 μL aliquot of the homogenised invertebrate mixtures could be aspirated with a micropipette (following the methods used in Poh et al. 2026). Water fleas were extracted directly after homogenisation without taking 10 μL aliquots, as they were very small (< 2 mm body size). We extracted DNA from 10 μL aliquots of homogenised invertebrate samples using PrepMan Ultra following the protocol established in Poh et al. (2026). DNA extracts were diluted 6:100 in molecular‐grade water before being used as templates in the PCR reaction. For each round of extraction, we also extracted a negative (a clean rayon‐tipped swab) and positive control (a swab dipped in *Bd* culture broth). Any leftover homogenised material was frozen at −20°C and kept for re‐extraction if further validation was required (as described below).

DNA extracts were analysed via qPCR following the protocol described above for amphibian skin swabs, including the standards and no template control wells, and all samples were run in singlicate. However, invertebrate samples often produce higher background noise within qPCR analysis (pers. obs), and false‐positive samples due to primer dimers are a risk when conducting qPCR assays to 40 cycles. Therefore, we decided to validate any positive invertebrate samples, to ensure that our positive samples were true‐positive results for *Bd* DNA presence. Invertebrate samples that were *Bd*‐positive, as determined by the shape of the amplification curve (exponential growth curve) in comparison with standards included on the same run. Initial qPCR screening of positive samples was validated by re‐running the extracted DNA in duplicate (such that the initial DNA extract for the positive qPCR well was run in triplicate in total). Because we used batch‐pooled samples that we homogenised in PBS, and we only extracted 10 μL of the insect slurry, we were able to conduct a second extraction on the same sample. For each batch‐pooled sample that was *Bd* positive, we went back to the original batch‐pooled sample, and extracted a second 10 μL of the insect slurry and analysed the second extracted sample via qPCR for *Bd* DNA presence in triplicate.

A sample was considered positive if *Bd* was detected in at least two qPCR wells (of the total of six qPCR extraction wells from two extractions of the same sample). We considered a sample negative if the first qPCR reaction was not positive for the presence of *Bd* DNA, but it is important to note that we are likely underestimating pathogen presence in these samples. Because the samples were batch‐pooled and homogenised, we expect any *Bd* present to be at low concentrations and challenging to detect in single samples. The invertebrate samples used in this study might be more similar to environmental DNA (eDNA) detection rather than frog samples, where DNA concentrations are also low that samples are often considered positive if 1 of 3 or 6 qPCR reaction wells are positive (Brannelly, Wetzel, Ohmer, et al. 2020b; Rees et al. 2014). However, to be conservative in reporting our results in this study, we considered samples where *Bd* DNA presence was detected in only one qPCR well of the six to be equivocal (not positive, but not negative). We included these equivocal results in the results section but did not include them in our overall prevalence estimates.

### 2.7 Statistical Analyses

This is an observational study to survey if *Bd* is present in freshwater invertebrates at sites with vulnerable amphibian populations. Therefore, our statistical analysis is descriptive, via calculating frequency and binomial confidence intervals in R Statistical Software (v4.4.2; R Core Team 2024). Confidence intervals were calculated using Clopper–Pearson exact method via the Hmisc R package (v5.2–3; Harrell 2025). We did not conduct statistical modelling in this study.

### 2.8 Animal Ethics and Scientific Permits

Frog sampling was conducted under approval from the Animal Ethics Committee at The University of Melbourne (Reference number 31053), and with the necessary research permits from the Department of Energy, Environment and Climate Action (Permit 10,011,340). The collection of invertebrates was authorised by the Department of Energy, Environment and Climate Action (Permit 10,011,199).

## 3 Results

### 3.1 Prevalence of Bd in Amphibians

**FIGURE 1:**
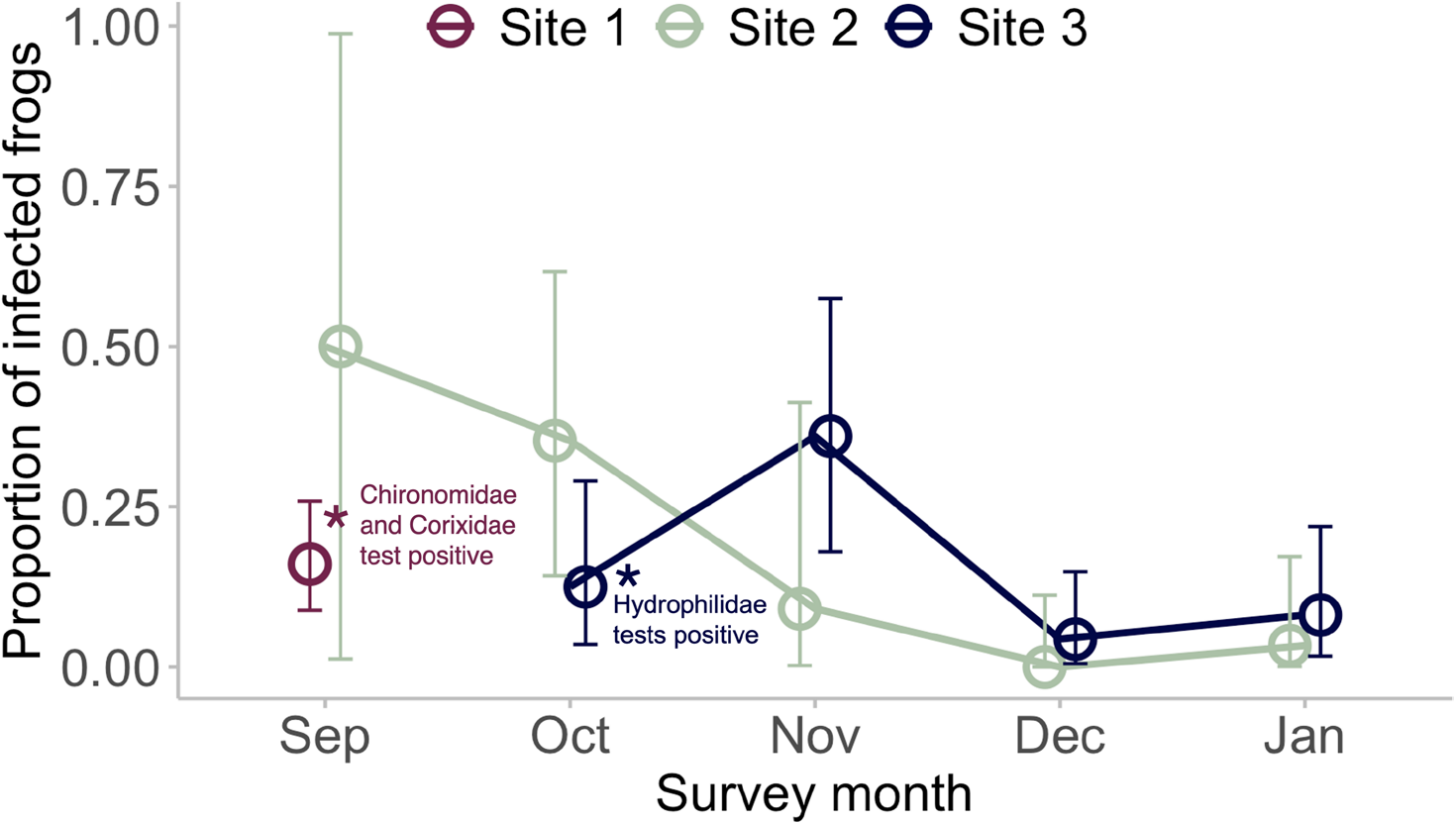
Proportion of frogs ( *L. aurea* or *L. raniformis* ) that tested positive for *Bd* presence based on qPCR testing of amphibian skin swabs from frogs captured at our collection sites and sampling timepoints. Points represent the proportion of samples at that timepoint that are positive, the error bars represent 95% confidence intervals, calculated using the Clopper–Pearson exact method based on a binomial distribution. Asterisks indicate that invertebrate samples collected from that site and timepoint tested positive for *Bd* DNA presence. Note that only two *L. raniformis* were collected at Site 2 in September; therefore, the prevalence is unreliable (and error bars are large).

Throughout the sampling period and at all sites, amphibian skin swabs collected from *Litoria* frogs tested positive for *Bd* (Figure 1) and 12.8% of all sampled frogs across the survey period (40 out of a total of 312 individual frogs) tested positive. At Site 1, which was only sampled in September, the proportion of frogs that tested positive for *Bd* was 0.16, 95% CI [0.00, 0.41]. For Sites 2 and 3 which were sampled over several months, the proportion of frogs that tested positive for *Bd* was the highest in October for Site 2 (0.35, 95% CI [0.14, 0.62]) and November for Site 3 (0.36, 95% CI [0.18, 0.57]).

### 3.2 Detection of Bd in Invertebrate Samples

Invertebrates across six different orders were collected and screened for *Bd* presence (Table S1). In total, 1015 individual invertebrates were extracted in 137 pooled batches. Four of these pooled samples were potential positive samples of *Bd* DNA, meaning that the first qPCR run was positive (Table S2). When we validated these four samples, we determined that three were true‐positive samples (two or more of the six qPCR reaction replicates were positive for *Bd* DNA), and one was an equivocal result (Figure 1; Table S2). Therefore, 2.1% of the batch‐pooled samples (3 of 137 invertebrate samples analysed) tested true positive for *Bd* DNA presence (Tables S1 and S2).

The positive and equivocal samples were found across three orders and three separate families of invertebrates, and found at two of the three surveyed sites (Figure 1, Table S1). The first sample that tested positive was a batch of adult flies from the family Chironomidae (non‐biting midges), collected from Site 1 in September (1 of 13 total batches of the Chironomidae family, and the sample contained 3 individuals). The second positive sample was a batch of adults from the family Corixidae (water boatmen) from Site 1 in September (1 of 19 total batches of the Corixidae family, and this batch contained 10 individuals). And third, larvae from the family Hydrophilidae (water scavenger beetles) collected from Site 3 in October tested positive for *Bd* (1 of 2 batches analysed from the Hydrophilidae family containing 5 individuals). A second batch from the same site and sampling period returned an equivocal result (containing 6 individuals).

We sampled Chironomidae larva and pupae (13 batches comprising a total of 81 individuals) and found no positive samples (Table S1). We also sampled adult Hydrophilidae (7 batches containing 43 individuals) and found no positive samples. The other taxa analysed that were negative for *Bd* DNA presence include Amphipoda (23 batches comprised of 153 individuals), Dysticidae (21 batches comprised 177 individuals, both adult and larvae), Notonectidae (15 batches comprised of 120 individuals), Zygoptera (8 batches of 56 individual larvae), Dolichopodidae (4 batches comprised of 33 individuals), Brachycera (3 batches comprised of 30 individuals), Daphnia (3 batches comprised of 30 individuals) and Anisotera (5 batches comprised of 18 individual larvae; Table S1).

## 4 Discussion

### 4.1 Diversity of Insects Positive for *Bd* DNA

We found three insect groups to be positive for *Bd* DNA and found an overall 2.1% prevalence of *Bd* within our pooled samples. Invertebrate samples from three major taxonomic orders within the class Insecta—*Diptera* (Family Chironomidae), *Coleoptera* (Family Hydrophilidae) and *Hemiptera* (Family Corixidae)—tested positive for *Bd*. To date, very little is known about insect associations with *Bd*. Of these three orders, Dipterans (flies) are the most studied in terms of their potential to act as *Bd* vectors. In field surveys, field‐caught midges from Brazil (*Corethrella* spp.) tested positive for *Bd* presence by qPCR, marking the only published account of wild‐caught insects testing positive for *Bd* (Toledo et al. 2021). Other Dipterans, including mosquitoes ( *Culex territans* and *C. quinquefasciatus* ) and fruit flies ( *Drosophila melanogaster* ) are able to act as carriers of *Bd* in laboratory experiments, but these studies could not confirm active infection nor the viability of *Bd* carried by exposed individuals, and no studies have demonstrated *Bd* presence in these species in the field (Byer et al. 2024; Gould et al. 2019; Reinhold et al. 2023). The vector potential of aquatic beetles (Order: Coleoptera) and water bugs (Order: Hemiptera) remains unstudied in published literature. Overall, very little is known about the presence of invertebrate carriers of *Bd* in Australia and the world.

To the best of our knowledge, the only record of *Bd* detections in freshwater invertebrates from Australia is in an unpublished Master's thesis (McNab 2015). McNab (2015) reported positive samples across many families of aquatic invertebrates from rainforest streams in Queensland, including mayflies (Order: Ephemeroptera), water‐pennies (Order: Coleoptera), damselflies (Order: Odonata), caddisflies (Order: Trichoptera) and water striders (Order: Hemiptera). Despite these astounding findings, the study was never published, and no research has subsequently attempted to build upon these findings in the last decade. Given the potential implications on amphibian conservation and the precedent of false positives in invertebrate detections (Kolby et al. 2015; Paulraj et al. 2016; Pessier et al. 2017; Rowley et al. 2007), it is critical that we continue to survey freshwater invertebrates for *Bd* to help us understand if non‐amphibian hosts might be important carriers or vectors of *Bd*.

The only other study in Australia that attempted to survey invertebrates for *Bd* was a field survey of Australian crayfish *Cherax destructor* in Kosciuszko National Park (Brannelly, Hunter, et al. 2015a). In the Australian Alps, *Bd* prevalence in frogs is high, but the reason for pathogen spread to these sites is unknown because it is remote with little human traffic. However, this survey did not find any *Bd*‐positive crayfish; therefore, *C. destructor* might not be an important vector of *Bd* (Brannelly, Hunter, et al. 2015a), particularly at high elevation sites in Australia. However, it is worth investigating the potential role of *C. destructor* at more sites, considering its importance in aquaculture that has facilitated its movement across the Australian landscape (Coughran et al. 2009). Rowley et al. (2006) originally detected *Bd* presence in freshwater shrimp (one *Caridina zebra* and two *Macrobrachium* spp. shrimp individuals) collected from rainforest streams in northern Queensland. However, an extensive sampling effort led by the same investigators the following year did not detect *Bd* in any shrimp collected from the same site (Rowley et al. 2007). Further, they were unable to experimentally infect *C. zebra* shrimp and *C. quadricarinatus* crayfish in the lab, leading them to believe that freshwater crustaceans might not carry *Bd* in northern Queensland, and that the previous detections in 2006 were false positives (Rowley et al. 2007). However, *Bd* is known to have heterogeneous distribution within and across species, and so it is certainly possible for there to be differential responses across populations and sampling years.

### 4.2 Chironomidae as Possible Vectors

The three groups of invertebrates that were *Bd*‐positive in our study have strong potential to be vectors of disease and could assist in the spread of this pathogen to novel sites. Chironomids (or non‐biting midges) are flies with aquatic egg, larval and pupal stages, and terrestrial flying adult stages. Although adults are non‐aquatic, they are found in high abundance in freshwater habitats, often swarming above water or nearby vegetation. While chironomids do not exhibit blood‐feeding behaviour, large swarms might facilitate the spread of *Bd* among amphibians through contact between chironomid individuals in a swarm and amphibians, or from infected surfaces like leaf litter, or infected waterbodies that they access. We expect transmission to be similar to the mechanical transmission facilitated by *Culex* mosquitoes in laboratory experiments (Gould et al. 2019; Reinhold et al. 2023). Chironomids at all life stages are known hosts of various microparasites, including some aquatic fungal parasites that are present outside their body (de Souza et al. 2014; Failla et al. 2015). A diverse range of viable fungal propagules were found in the gastrointestinal tracts of invasive chironomid larvae ( *Eretmoptera murphyi* ) in Antarctica, raising concerns that invasive chironomids might act as vectors for fungal pathogens (Bridge and Denton 2007). While chironomid adults are not strong fliers, wind can aid with dispersing chironomid adults across long distances to new habitats (Bitušík et al. 2017; Bohonak and Jenkins 2003). Invasions of chironomids have also been facilitated by shipping, airplane stowaways and soil movement, although many chironomid invasions have no known cause (Failla et al. 2015). Chironomids rapidly colonise man‐made aquatic infrastructure (Failla et al., 2015; Hribar et al. 2008), which is of particular concern for artificial ponds used as *Bd*‐free havens in captive‐breeding efforts (Office of Environment and Heritage 2013). Considering their swarming behaviour, ability to disperse long distances, invasive potential and the existence of a variety of known fungal parasites, our study's detection of *Bd* in field‐caught adult chironomids is highly concerning. Though it should be noted that the larval individuals tested in our study were not positive for *Bd* thus spread through the water by chironomids is not a mechanism of pathogen spread that we are proposing here.

### 4.3 Hydrophilidae as Possible Vectors

Water scavenger beetles (Hydrophilidae) are one of the most speciose families of aquatic Coleoptera and are commonly found in stagnant or slow‐moving freshwater habitats. Most species are aquatic throughout both larval and adult stages. Adults primarily feed on plants or decaying organic matter, while larvae are carnivorous (Gooderham and Tsyrlin 2002; Yee and Kehl 2015). They are known hosts to many species of aquatic parasitic fungi from the order Laboulbeniales (Calabon et al. 2023). In this study, *Bd* was only detected in larval samples: one positive and one equivocal sample. We found no positive samples among the adult hydrophilids tested. However, adult sample sizes were low at each site and timepoint, particularly at Site 3 where larval positive and equivocal samples were found, limiting conclusions about the vector potential of adult hydrophilids. Low sample sizes lead to large confidence intervals for disease prevalence analysis, which means we cannot be confident that the adult water scavenger beetles are not potential carriers of *Bd*. Furthermore, we do not know if larvae can carry infection or carry infection through to adulthood. Adults are potentially of greater interest when considering disease spread, as they can fly between water bodies. But the aquatic larval stage co‐habits with amphibians and could lead to pathogen spread through the water. It is important to continue surveying this group for *Bd*‐positive adults and larvae to understand the risk they pose as potential vectors of chytridiomycosis.

### 4.4 Corixidae as Possible Vectors

Corixidae (water boatmen) is a cosmopolitan family of aquatic true bugs commonly found in high abundance in still or slow‐flowing waters. Corixids are concerning as potential vectors of *Bd* because of their high abundance and ability to disperse through flight and human activities such as shipping and thus have a risk of becoming invasive (Gooderham and Tsyrlin 2002). Corixidae contains one of the few examples of a highly invasive fully aquatic insect: *Trichocorixa verticalis verticalis* . *T. v. verticalis* is invasive in three continents and has spread beyond its native range in North America likely in ballast water through shipping (Carbonell et al. 2016; Fenoglio et al. 2016; Ortego et al. 2021). Corixids are natural hosts for fungal parasites from the genus *Coreomyces* (Order Laboulbeniales, Phylum Ascomycota) (Sundberg et al. 2021) and one species has also shown susceptibility to infection by *Metarhizium* fungi (Order Hypocreales, Phylum Ascomycota) in laboratory experiments (Belevich et al. 2017). Therefore, the ecology, potential of movement across the landscape and possible host of fungal pathogens warrants a further look into the vector potential of corixids to harbour *Bd*.

### 4.5 Detection in Invertebrates Coincided With Detection in Amphibians

We confirmed *Bd* presence at all three of our sites throughout our sampling period through detections of *Bd*‐positive amphibian skin swabs taken from *Litoria* frogs. The overall amphibian *Bd* prevalence across the sampling period was 12.8% (40 out of 312 sampled frogs). The decrease in infection prevalence in December and January across all sites coincides with summer in Victoria, aligning with known seasonal patterns of *Bd* infections in wild amphibian populations (Berger et al. 2004; Brannelly et al. 2018; Kriger and Hero 2007). The prevalence of *Bd* infection tends to be lowest during warm summer months due to the low heat tolerance of the fungus (Berger et al. 2004; Kriger and Hero 2007). The overall prevalence of *Bd* in invertebrates that we found was lower than the prevalence in the sampled amphibians, but this low prevalence is likely high enough to maintain the pathogen within the system. Disease reservoirs or intermediate hosts of pathogens often have much lower prevalence than susceptible species in the field, some having an infection prevalence of < 2% (e.g., snail hosts with *Fasciola hepatica* , Kaplan et al. 1997). This low *Bd* DNA detection is similar to the few other invertebrate surveys that have been conducted, which also found prevalence in invertebrates to be lower than the amphibians at the same sites (Brannelly, McMahon, et al. 2015b; McMahon et al. 2013). Sampling invertebrates might seem preferable to sampling amphibians for disease detection, because animal ethics are usually not required and sampling permits are easier to obtain, but prevalence is likely to be low in invertebrates, and importantly, negative samples will likely not indicate a negative site. This low prevalence is similar to using eDNA as a tool to test for *Bd* presence. eDNA can be helpful for detecting pathogen presence at a site but cannot accurately determine disease dynamics or pathogen absence from a site because it is not as sensitive as direct amphibian sampling (Brannelly, Wetzel, Ohmer, et al. 2020b).

### 4.6 Limitations of Screening Invertebrates Using Molecular Methods

Our detection of *Bd* in some field‐caught invertebrates from the families Chironomidae, Hydrophilidae and Corixidae highlights these groups as promising candidates for further vector competence studies. However, taxa that tested negative should not be dismissed as potential vectors. Invertebrate samples that returned negative results in the initial screening might be missed detections, in part due to the batch pooling method we employed that allowed us to survey more invertebrate individuals over broader taxa than if we extracted samples individually, and ran the qPCR reaction for each sample in triplicate (Brannelly, Wetzel, West, and Richards‐Zawacki 2020a). However, we expect invertebrate samples to contain low *Bd* loads overall due to both their size, and previous research indicating that loads are lower than what we would expect on frogs (Brannelly, McMahon, et al. 2015b; McMahon et al. 2013; Toledo et al. 2021). Low DNA loads in samples is a common issue with studies attempting to detect *Bd* DNA from environmental samples (Brannelly, Wetzel, Ohmer, et al. 2020b). For eDNA studies, it is recommended to take several samples at each collection timepoint, and return to sites several times in order to increase the probability of detecting *Bd* DNA (Brannelly, Wetzel, Ohmer, et al. 2020b). A similar procedure could be used for detecting *Bd* in invertebrate samples to increase the chances of detecting *Bd* within the sample. There are more sensitive genetic methods for detecting pathogen DNA, like digital PCR (dPCR) that have been used for *Bd* detection (Everts et al. 2025; Porco et al. 2024) and could be employed in future invertebrate vectors studies where pathogen load is expected to be low. While dPCR are often used in studies to genotype the pathogen (Jenkinson et al. 2018; Ribeiro et al. 2025), they have been used for pathogen detection of *Bd* in eDNA samples (Everts et al. 2025; Porco et al. 2024) and could be useful in invertebrate samples.

We followed strict hygiene protocols for sampling and molecular techniques, and all of our negative controls were negative for *Bd*. We are confident our *Bd* positive samples are not false positives. We also validated our positive samples by requiring at least two positive qPCR wells. Nevertheless, we would like to stress the importance of continued surveillance of invertebrates in the field for *Bd* presence to identify candidate taxa that might have been missed in this study and reproduce our findings regarding *Bd*‐positive taxa.

Positive qPCR results are unable to confirm active infection or viability of *Bd* on invertebrate samples. Further vector competence studies, such as those conducted by McMahon et al. (2013) on crayfish, are necessary to confirm that the invertebrate taxa found positive in this study can function as vectors. These include laboratory infection experiments, with active infection verified through examination of histological sections or fungal culture from infected individuals, and transmission experiments investigating transmission of *Bd* from invertebrates to disease‐free amphibians. Establishing any species as a competent pathogen vector requires multiple lines of evidence. For example, mosquitoes were only confirmed as vectors of Buruli ulcer in humans after PCR‐based field detections were supported with experimental transmission studies with mice, spatial modelling and various genomic analyses (Mee et al. 2024; Merritt et al. 2010; Wallace et al. 2017). A similarly rigorous approach is needed to evaluate the vector potential of non‐amphibian hosts for *Bd*, ensuring conclusions are supported by both experimental and field data.

## 5 Conclusion

Our study detected *Bd* DNA in three new invertebrate families. While these findings should be conservatively interpreted until experimental infection and transmission capability is confirmed in these groups, this represents an important first step in narrowing the pool of candidate taxa for future vector competence research. Discovering capable invertebrate vectors might have major implications for biosecurity measures, necessitating the need to account for the movement of identified freshwater invertebrate vectors (e.g., through ballast water management). There are also implications for captive‐breeding and reintroduction programmes, which might benefit from implementing measures to exclude wild invertebrate vectors from disease‐free enclosures. The detection of *Bd* across three separate orders (Hemiptera, Coleoptera and Diptera) indicates that *Bd* associations with invertebrates might be more taxonomically widespread than previously recognised. This underscores how little is currently known about the role of non‐amphibian organisms in the spread of chytridiomycosis and highlights the need for further research in this area to inform conservation strategies. As chytridiomycosis continues to drive global amphibian declines decades after its discovery, understanding the role of non‐amphibian organisms in disease dynamics might prove critical. We encourage continued research in this area and emphasise the importance of publishing negative as well as positive results. Identifying which non‐amphibian taxa do not carry or transmit *Bd* is just as important as identifying those that do, yet such findings are often underreported due to publication bias (McMahon et al. 2021; Mlinarić et al. 2017). A more complete understanding of potential non‐amphibian vectors is essential to protect remaining amphibian biodiversity.

## Author Contributions

**Amanda Poh:** conceptualization (equal), data curation (equal), formal analysis (equal), investigation (equal), methodology (equal), writing – original draft (equal). **Perran A. Ross:** conceptualization (equal), project administration (equal), supervision (equal), writing – review and editing (equal). **Venice Chan:** investigation (equal), writing – review and editing (equal). **Taegan McMahon:** conceptualization (equal), methodology (equal), writing – review and editing (equal). **Caitlin L. Nordheim‐Maestas:** investigation (equal), writing – review and editing (equal). **Laura A. Brannelly:** conceptualization (equal), data curation (equal), formal analysis (equal), investigation (equal), methodology (equal), project administration (equal), resources (equal), supervision (lead), validation (equal), writing – review and editing (equal).

## Funding

This work was supported by the Australian Research Council, DE230100067.

## Conflicts of Interest

The authors declare no conflicts of interest.

## Supporting information


**Table S1:** Invertebrate samples collected and processed and where *Bd* DNA was detected within the samples
**Table S2:** The Bd DNA load that was detected within the positive samples

## Data Availability

Data is presented in Appendix 1: Tables S1 and S2.

## References

[ece373513-bib-0001] Becker, C. G. , and K. R. Zamudio . 2011. “Tropical Amphibian Populations Experience Higher Disease Risk in Natural Habitats.” Proceedings of the National Academy of Sciences of the United States of America 108: 9893–9898. 10.1073/pnas.1014497108. 21628560 PMC3116417

[ece373513-bib-0002] Belevich, O. E. , Y. A. Yurchenko , V. V. Glupov , and V. Y. Kryukov . 2017. “Effect of Entomopathogenic Fungus *Metarhizium robertsii* on Non‐Target Organisms, Water Bugs (Heteroptera: Corixidae, Naucoridae, Notonectidae).” Applied Entomology and Zoology 52: 439–445. 10.1007/s13355-017-0494-z.

[ece373513-bib-0003] Berger, L. , R. Speare , H. B. Hines , et al. 2004. “Effect of Season and Temperature on Mortality in Amphibians due to Chytridiomycosis.” Australian Veterinary Journal 82: 434–439. 10.1111/j.1751-0813.2004.tb11137.x. 15354853

[ece373513-bib-0004] Bitušík, P. , M. Svitok , M. Novikmec , K. Trnkova , and L. Hamerlík . 2017. “A Unique Way of Passive Dispersal of Aquatic Invertebrates by Wind: Chironomid Larvae Are Traveling in Fragments of Aquatic Mosses.” Limnologica ‐ Ecology and Management of Inland Waters 63: 119–121. 10.1016/j.limno.2017.02.001.

[ece373513-bib-0005] Bletz, M. C. , G. M. Rosa , F. Andreone , et al. 2015. “Widespread Presence of the Pathogenic Fungus *Batrachochytrium dendrobatidis* in Wild Amphibian Communities in Madagascar.” Scientific Reports 5: 8633. 10.1038/srep08633. 25719857 PMC4341422

[ece373513-bib-0006] Bohonak, A. J. , and D. G. Jenkins . 2003. “Ecological and Evolutionary Significance of Dispersal by Freshwater Invertebrates.” Ecology Letters 6: 783–796. 10.1046/j.1461-0248.2003.00486.x.

[ece373513-bib-0007] Bower, D. S. , K. R. Lips , Y. Amepou , et al. 2019. “Island of Opportunity: Can New Guinea Protect Amphibians From a Globally Emerging Pathogen?” Frontiers in Ecology and the Environment 17: 348–354. 10.1002/fee.2057.

[ece373513-bib-0008] Boyle, D. G. , D. B. Boyle , V. Olsen , J. A. T. Morgan , and A. D. Hyatt . 2004. “Rapid Quantitative Detection of Chytridiomycosis (*Batrachochytrium dendrobatidis*) in Amphibian Samples Using Real‐Time Taqman PCR Assay.” Diseases of Aquatic Organisms 60: dao060141. 10.3354/dao060141. 15460858

[ece373513-bib-0009] Brannelly, L. A. , M. W. H. Chatfield , J. Sonn , M. Robak , and C. L. Richards‐Zawacki . 2018. “Fungal Infection Has Sublethal Effects in a Lowland Subtropical Amphibian Population.” BMC Ecology 18: 34. 10.1186/s12898-018-0189-5. 30217158 PMC6137908

[ece373513-bib-0010] Brannelly, L. A. , D. A. Hunter , D. Lenger , B. C. Scheele , L. F. Skerratt , and L. Berger . 2015a. “Dynamics of Chytridiomycosis During the Breeding Season in an Australian Alpine Amphibian.” PLoS One 10: e0143629. 10.1371/journal.pone.0143629. 26629993 PMC4668081

[ece373513-bib-0011] Brannelly, L. A. , T. A. McMahon , M. Hinton , D. Lenger , and C. L. Richards‐Zawacki . 2015b. “ *Batrachochytrium dendrobatidis* in Natural and Farmed Louisiana Crayfish Populations: Prevalence and Implications.” Diseases of Aquatic Organisms 112: dao02817. 10.3354/dao02817. 25590773

[ece373513-bib-0012] Brannelly, L. A. , D. Wetzel , M. West , and C. L. Richards‐Zawacki . 2020a. “Optimized Swab Sample Extraction Results in Imperfect Detection of *Batrachochytrium dendrobatidis* Particularly When Infection Intensities Are Low.” Diseases of Aquatic Organisms 139: dao03482. 10.3354/dao03482. 32495749

[ece373513-bib-0013] Brannelly, L. A. , D. P. Wetzel , M. E. B. Ohmer , L. Zimmerman , V. Saenz , and C. L. Richards‐Zawacki . 2020b. “Evaluating Environmental DNA as a Tool for Detecting an Amphibian Pathogen Using an Optimized Extraction Method.” Oecologia 194: 267–281. 10.1007/s00442-020-04743-4. 32880026

[ece373513-bib-0014] Bridge, P. D. , and G. J. Denton . 2007. “Isolation of Diverse Viable Fungi From the Larvae of the Introduced Chironomid *eretmoptera murphyi* on Signy Island.” Polar Biology 30: 935–937. 10.1007/s00300-007-0268-0.

[ece373513-bib-0015] Burrowes, P. A. , and I. De la Riva . 2017. “Detection of the Amphibian Chytrid Fungus *Batrachochytrium dendrobatidis* in Museum Specimens of Andean Aquatic Birds: Implications for Pathogen Dispersal.” Journal of Wildlife Diseases 53: 349–355. 10.7589/2016-04-074. 28094607

[ece373513-bib-0016] Byer, A. M. , K. M. Nguyen , T. S. Katz , R. Chen , and C. J. Briggs . 2024. “ *Drosophila melanogaster* As a Model Arthropod Carrier for the Amphibian Chytrid Fungus *Batrachochytrium dendrobatidis* .” PLoS One 19: e0307833. 10.1371/journal.pone.0307833. 39047007 PMC11268706

[ece373513-bib-0017] Calabon, M. , K. Hyde , E. Jones , et al. 2023. “Freshwater Fungal Biology.” Mycosphere 14: 195–413. 10.5943/mycosphere/14/1/4.

[ece373513-bib-0018] Carbonell, J. A. , A. Millán , A. J. Green , V. Céspedes , C. Coccia , and J. Velasco . 2016. “What Traits Underpin the Successful Establishment and Spread of the Invasive Water Bug *Trichocorixa verticalis verticalis* ?” Hydrobiologia 768: 273–286. 10.1007/s10750-015-2556-y.

[ece373513-bib-0019] Coughran, J. , R. Mccormack , and G. Daly . 2009. “Translocation of the Yabby *Cherax destructor* Into Eastern Drainages of New South Wales, Australia.” Australian Zoologist 35: 100–103. 10.7882/AZ.2009.009.

[ece373513-bib-0020] de Souza, J. I. , F. H. Gleason , M. A. Ansari , et al. 2014. “Fungal and Oomycete Parasites of *Chironomidae*, *Ceratopogonidae* and *Simuliidae* (*Culicomorpha*, *Diptera*).” Fungal Biology Reviews 28: 13–23. 10.1016/j.fbr.2014.02.002.

[ece373513-bib-0021] Department of the Environment and Energy . 2016. “Threat abatement plan for infection of amphibians with chytrid fungus resulting in chytridiomycosis. Australian Government.”

[ece373513-bib-0022] Everts, T. , C. Van Driessche , H. Jacquemyn , S. Neyrinck , D. Halfmaerten , and R. Brys . 2025. “Is Exposure to Chytrid Fungus and Ranavirus Higher in Ponds Invaded by American Bullfrogs?” Biological Invasions 27: 193. 10.1007/s10530-025-03648-8.

[ece373513-bib-0023] Failla, A. J. , A. A. Vasquez , M. Fujimoto , and J. L. Ram . 2015. “The Ecological, Economic and Public Health Impacts of Nuisance Chironomids and Their Potential as Aquatic Invaders.” Aquatic Invasions 10: 1–15. 10.3391/ai.2015.10.1.01.

[ece373513-bib-0024] Fenoglio, S. , N. Bonada , S. Guareschi , M. J. López‐Rodríguez , A. Millán , and J. M. Tierno De Figueroa . 2016. “Freshwater Ecosystems and Aquatic Insects: A Paradox in Biological Invasions.” Biology Letters 12: 20151075. 10.1098/rsbl.2015.1075. 27072403 PMC4881343

[ece373513-bib-0025] Fisher, M. C. , and T. W. J. Garner . 2007. “The Relationship Between the Emergence of *Batrachochytrium dendrobatidis*, the International Trade in Amphibians and Introduced Amphibian Species.” Fungal Biology Reviews 21: 2–9. 10.1016/j.fbr.2007.02.002.

[ece373513-bib-0026] Fisher, M. C. , T. W. J. Garner , and S. F. Walker . 2009. “Global Emergence of *Batrachochytrium dendrobatidis* and Amphibian *Chytridiomycosis* in Space, Time, and Host.” Annual Review of Microbiology 63: 291–310. 10.1146/annurev.micro.091208.073435. 19575560

[ece373513-bib-0027] Garmyn, A. , P. Van Rooij , F. Pasmans , et al. 2012. “Waterfowl: Potential Environmental Reservoirs of the Chytrid Fungus *Batrachochytrium dendrobatidis* .” PLoS One 7: e35038. 10.1371/journal.pone.0035038. 22514705 PMC3325947

[ece373513-bib-0028] Gooderham, J. , and E. Tsyrlin . 2002. The Waterbug Book: A Guide to the Freshwater Macroinvertebrates of Temperature Australia. CSIRO Publishing.

[ece373513-bib-0029] Gould, J. , J. W. Valdez , M. P. Stockwell , S. Clulow , and M. J. Mahony . 2019. “Mosquitoes as a Potential Vector for the Transmission of the Amphibian Chytrid Fungus.” Zoology and Ecology 29: 36–42. 10.35513/21658005.2019.1.5.

[ece373513-bib-0030] Harrell, F. E. 2025. “Hmisc: Harrell Miscellaneous. R package version 5.2‐3.” URL https://CRAN.R‐project.org/package=Hmisc.

[ece373513-bib-0031] Heard, G. W. , M. P. Scroggie , and B. S. Malone . 2012. “The Life History and Decline of the Threatened Australian Frog, *Litoria raniformis* .” Austral Ecology 37: 276–284. 10.1111/j.1442-9993.2011.02275.x.

[ece373513-bib-0032] Hribar, L. J. , J. H. Epler , J. Martin , and J. E. Sublette . 2008. “ *Chironomus columbiensis* (*Diptera*: *chironomidae*) New to the Fauna of the United States.” Florida Entomologist 91: 470–471. 10.1653/0015-4040(2008)91[470:CCDCNT]2.0.CO;2.

[ece373513-bib-0033] Jenkinson, T. S. , D. Rodriguez , R. A. Clemons , et al. 2018. “Globally Invasive Genotypes of the Amphibian Chytrid Outcompete an Enzootic Lineage in Coinfections.” Proceedings of the Royal Society B: Biological Sciences 285: 20181894. 10.1098/rspb.2018.1894. PMC6304064 30963903

[ece373513-bib-0034] Johnson, M. L. , and R. Speare . 2005. “Possible Modes of Dissemination of the Amphibian Chytrid *Batrachochytrium dendrobatidis* in the Environment.” Diseases of Aquatic Organisms 65: dao065181. 10.3354/dao065181. 16119886

[ece373513-bib-0035] Kaplan, R. M. , J. B. Dame , G. R. Reddy , and C. H. Courtney . 1997. “The Prevalence of *Fasciola hepatica* in Its Snail Intermediate Host Determined by DNA Probe Assay.” International Journal for Parasitology 27: 1585–1593. 10.1016/S0020-7519(97)00139-2. 9467746

[ece373513-bib-0036] Kilburn, V. L. , R. Ibáñez , and D. M. Green . 2011. “Reptiles as Potential Vectors and Hosts of the Amphibian Pathogen *Batrachochytrium dendrobatidis* in Panama.” Diseases of Aquatic Organisms 97: dao02409. 10.3354/dao02409. 22303629

[ece373513-bib-0037] Kolby, J. E. 2014. “Presence of the Amphibian Chytrid Fungus *Batrachochytrium dendrobatidis* in Native Amphibians Exported From Madagascar.” PLoS One 9: e89660. 10.1371/journal.pone.0089660. 24599336 PMC3943771

[ece373513-bib-0038] Kolby, J. E. , K. M. Smith , S. D. Ramirez , et al. 2015. “Rapid Response to Evaluate the Presence of Amphibian Chytrid Fungus (*Batrachochytrium dendrobatidis*) and Ranavirus in Wild Amphibian Populations in Madagascar.” PLoS One 10: e0125330. 10.1371/journal.pone.0125330. 26083349 PMC4471163

[ece373513-bib-0039] Kriger, K. M. , and J. M. Hero . 2007. “Large‐Scale Seasonal Variation in the Prevalence and Severity of Chytridiomycosis.” Journal of Zoology 271: 352–359. 10.1111/j.1469-7998.2006.00220.x.

[ece373513-bib-0040] Kriger, K. M. , and J. M. Hero . 2009. “Chytridiomycosis, Amphibian Extinctions, and Lessons for the Prevention of Future Panzootics.” EcoHealth 6: 6–10. 10.1007/s10393-009-0228-y. 19421815

[ece373513-bib-0041] Lindenmayer, D. , and B. C. Scheele . 2017. “Do Not Publish.” Science 356: 800–801. 10.1126/science.aan1362. 28546170

[ece373513-bib-0042] McMahon, T. A. , L. A. Brannelly , M. W. H. Chatfield , et al. 2013. “Chytrid Fungus *Batrachochytrium dendrobatidis* Has Nonamphibian Hosts and Releases Chemicals That Cause Pathology in the Absence of Infection.” Proceedings of the National Academy of Sciences of the United States of America 110: 210–215. 10.1073/pnas.1200592110. 23248288 PMC3538220

[ece373513-bib-0043] McMahon, T. A. , C. L. Nordheim , and D. M. Prokopiak . 2021. “Freshwater Snails and the Green Algae Cladophora Are Probably Not Hosts of *Batrachochytrium dendrobatidis* .” Freshwater Biology 66: 582–586. 10.1111/fwb.13662. 41050983 PMC12494061

[ece373513-bib-0044] McNab, A. 2015. “Dynamics of the amphibian chytrid fungus (*Batrachochytrium dendrobatidis*) in isolated patches of lowland rainforest. (MSc Thesis). James Cook University.” 10.25903/pn47-dd72.

[ece373513-bib-0045] Mee, P. T. , A. H. Buultjens , J. Oliver , et al. 2024. “Mosquitoes Provide a Transmission Route Between Possums and Humans for Buruli Ulcer in Southeastern Australia.” Nature Microbiology 9: 377–389. 10.1038/s41564-023-01553-1. PMC10847040 38263454

[ece373513-bib-0046] Merritt, R. W. , E. D. Walker , P. L. C. Small , et al. 2010. “Ecology and Transmission of Buruli Ulcer Disease: A Systematic Review.” PLoS Neglected Tropical Diseases 4: e911. 10.1371/journal.pntd.0000911. 21179505 PMC3001905

[ece373513-bib-0047] Mlinarić, A. , M. Horvat , and V. Šupak Smolčić . 2017. “Dealing With the Positive Publication Bias: Why You Should Really Publish Your Negative Results.” Biochemia Medica 27: 030201. 10.11613/BM.2017.030201. 29180912 PMC5696751

[ece373513-bib-0048] Monzon, F. C. , M. O. Rödel , and J. M. Jeschke . 2020. “Tracking *Batrachochytrium dendrobatidis* Infection Across the Globe.” EcoHealth 17: 270–279. 10.1007/s10393-020-01504-w. 33201333 PMC7719156

[ece373513-bib-0049] Murray, K. , R. Retallick , K. R. McDonald , et al. 2010. “The Distribution and Host Range of the Pandemic Disease Chytridiomycosis in Australia, Spanning Surveys From 1956–2007.” Ecology 91: 1557–1558. 10.1890/09-1608.1.

[ece373513-bib-0050] Murray, K. , L. F. Skerratt , G. Marantelli , et al. 2011. “Hygiene protocols for the control of diseases in Australian frogs. (A report for the Australian Department of Sustainability, Environment, Water, Population and Communities).”

[ece373513-bib-0051] Office of Environment and Heritage . 2013. “Saving our species: Iconic species project ‐ Southern corroboree frog. NSW Government.”

[ece373513-bib-0052] Ortego, J. , V. Céspedes , A. Millán , and A. J. Green . 2021. “Genomic Data Support Multiple Introductions and Explosive Demographic Expansions in a Highly Invasive Aquatic Insect.” Molecular Ecology 30: 4189–4203. 10.1111/mec.16050. 34192379

[ece373513-bib-0053] Osborne, W. S. , M. J. Littlejohn , and S. A. Thomson . 2014. “Former Distribution and Apparent Disappearance of the *Litoria aurea* Complex From the Southern Tablelands of New South Wales and the Australian Capital Territory.” Australian Zoologist 30: 190–198. 10.7882/AZ.1996.011.

[ece373513-bib-0054] Paulraj, A. , M. S. Musthafa , K. Altaff , et al. 2016. “Chytrid *Batrachochytrium dendrobatidis* Fungal Infection in Freshwater Prawn, *Macrobrachium rosenbergii* (de Man) ‐ A New Report.” Aquaculture 464: 521–528. 10.1016/j.aquaculture.2016.07.035.

[ece373513-bib-0055] Pessier, A. P. , M. J. Forzán , J. E. Longcore , L. Berger , L. Rollins‐Smith , and L. F. Skerratt . 2017. “Letter to the Editor: Comment on Chytrid *Batrachochytrium dendrobatidis* Fungal Infection in Freshwater Prawn, *Macrobrachium rosenbergii* (de Man)‐a New Report.” Aquaculture 468: 326–327. 10.1016/j.aquaculture.2016.10.026.

[ece373513-bib-0056] Poh, A. , P. Ross , and L. Brannelly . 2026. “Cost‐Effective DNA Extraction Method for Detection of Amphibian Fungal Pathogen in Invertebrate Samples.” Diseases of Aquatic Organisms 165: dao03905. 10.3354/dao03905. 41817029

[ece373513-bib-0057] Porco, D. , C. A. Purnomo , L. Glesener , et al. 2024. “eDNA‐Based Monitoring of *Batrachochytrium dendrobatidis* and *Batrachochytrium salamandrivorans* With ddPCR in Luxembourg Ponds: Taking Signals Below the Limit of Detection (LOD) Into Account.” BMC Ecology and Evolution 24: 4. 10.1186/s12862-023-02189-9. 38178008 PMC10768104

[ece373513-bib-0058] R Core Team . 2024. R: A Language and Environment for Statistical Computing. R Foundation for Statistical Computing.

[ece373513-bib-0059] Rees, H. C. , B. C. Maddison , D. J. Middleditch , J. R. M. Patmore , and K. C. Gough . 2014. “REVIEW: The Detection of Aquatic Animal Species Using Environmental DNA – A Review of eDNA as a Survey Tool in Ecology.” Journal of Applied Ecology 51: 1450–1459. 10.1111/1365-2664.12306.

[ece373513-bib-0060] Reinhold, J. M. , E. Halbert , M. Roark , et al. 2023. “The Role of *Culex territans* Mosquitoes in the Transmission of *Batrachochytrium dendrobatidis* to Amphibian Hosts.” Parasites & Vectors 16: 424. 10.1186/s13071-023-05992-x. 37974288 PMC10655354

[ece373513-bib-0061] Ribeiro, L. P. , D. Rodriguez , R. C. dos Santos , E. M. Lucas , and L. F. Toledo . 2025. “Genotypic Discrimination of Chytrid Fungus Lineages in the Amphibian Trade.” EcoHealth 22: 553–564. 10.1007/s10393-025-01736-8. 40736633

[ece373513-bib-0062] Rohr, J. R. , N. T. Halstead , and T. R. Raffel . 2011. “Modelling the Future Distribution of the Amphibian Chytrid Fungus: The Influence of Climate and Human‐Associated Factors.” Journal of Applied Ecology 48: 174–176. 10.1111/j.1365-2664.2010.01891.x.

[ece373513-bib-0063] Rosenblum, E. B. , T. Y. James , K. R. Zamudio , et al. 2013. “Complex History of the Amphibian‐Killing Chytrid Fungus Revealed With Genome Resequencing Data.” Proceedings of the National Academy of Sciences of the United States of America 110: 9385–9390. 10.1073/pnas.1300130110. 23650365 PMC3677446

[ece373513-bib-0064] Rowley, J. J. L. , R. A. Alford , and L. F. Skerratt . 2006. “The Amphibian Chytrid *Batrachochytrium dendrobatidis* Occurs on Freshwater Shrimp in Rain Forest Streams in Northern Queensland, Australia.” EcoHealth 3: 49–52. 10.1007/s10393-005-0005-5.

[ece373513-bib-0065] Rowley, J. J. L. , V. Hemingway , R. Alford , et al. 2007. “Experimental Infection and Repeat Survey Data Indicate the Amphibian Chytrid *Batrachochytrium dendrobatidis* May Not Occur on Freshwater Crustaceans in Northern Queensland, Australia.” EcoHealth 4: 31–36. 10.1007/s10393-006-0075-z.

[ece373513-bib-0066] Scheele, B. C. , F. Pasmans , L. F. Skerratt , et al. 2019. “Amphibian Fungal Panzootic Causes Catastrophic and Ongoing Loss of Biodiversity.” Science 363: 1459–1463. 10.1126/science.aav0379. 30923224

[ece373513-bib-0067] Scheele, B. C. , L. F. Skerratt , L. F. Grogan , et al. 2017. “After the Epidemic: Ongoing Declines, Stabilizations and Recoveries in Amphibians Afflicted by Chytridiomycosis.” Biological Conservation 206: 37–46. 10.1016/j.biocon.2016.12.010.

[ece373513-bib-0068] Shapard, E. J. , A. S. Moss , and M. J. San Francisco . 2012. “ *Batrachochytrium dendrobatidis* Can Infect and Cause Mortality in the Nematode *Caenorhabditis elegans* .” Mycopathologia 173: 121–126. 10.1007/s11046-011-9470-2. 22002554

[ece373513-bib-0069] Sundberg, H. , Å. Kruys , J. Bergsten , and S. Ekman . 2021. “ *Coreomyces* (*Laboulbeniales*) in Sweden, With Two New Species.” Nordic Journal of Botany 39: e03323. 10.1111/njb.03323.

[ece373513-bib-0070] Toledo, L. F. , J. Ruggeri , L. Leite Ferraz de Campos , M. Martins , S. Neckel‐Oliveira , and C. P. B. Breviglieri . 2021. “Midges Not Only Sucks, but May Carry Lethal Pathogens to Wild Amphibians.” Biotropica 53: 722–725. 10.1111/btp.12928.

[ece373513-bib-0071] Wallace, J. R. , K. M. Mangas , J. L. Porter , et al. 2017. “ *Mycobacterium ulcerans* Low Infectious Dose and Mechanical Transmission Support Insect Bites and Puncturing Injuries in the Spread of Buruli Ulcer.” PLoS Neglected Tropical Diseases 11: e0005553. 10.1371/journal.pntd.0005553. 28410412 PMC5406025

[ece373513-bib-0072] Webb, R. , D. Mendez , L. Berger , and R. Speare . 2007. “Additional Disinfectants Effective Against the Amphibian Chytrid Fungus *Batrachochytrium dendrobatidis* .” Diseases of Aquatic Organisms 74: 13–16. 17425259 10.3354/dao074013

[ece373513-bib-0073] Yee, D. A. , and S. Kehl . 2015. “Order Coleoptera.” In Thorp and Covich's Freshwater Invertebrates, edited by J. H. Thorp and M. B. A. Rogers , 1003–1042. Elsevier. 10.1016/B978-0-12-385026-3.00039-5.

