# Supplementary material for: Field Survey of Freshwater Invertebrates Reveals That Several Groups Are Potential Carriers of the Fungal Pathogen Batrachochytrium dendrobatidis
Source: Ecol Evol. 2026 Apr 27;16(4):e73513. doi: 10.1002/ece3.73513 (PMC13112068; doi:10.1002/ece3.73513)
Supplement: Supplementary file 1 — Table S1: Invertebrate samples collected and processed and where Bd DNA was detected within the samples Table S2: The Bd DNA load that was detected within the positive samples [file ECE3-16-e73513-s001.docx]

**Appendix 1**

Supplemental Tables

Table S1 – Invertebrate samples collected and processed and where *Bd* DNA was detected within the samples

Table S2 – The *Bd* DNA load that was detected within the positive samples

Table S1| Detection of *Bd* presence in invertebrates sampled from Sites 1 – 3 sampled September 2024 – January 2025. Invertebrates are grouped according to order, and identified to is the lowest taxonomic group that samples could be identified to. Life stage is indicated in brackets. Where life stage is not indicated, invertebrates were hemimetabolous and nymphs could not be readily distinguishable from adults. Groups where samples tested positive for *Bd* are in bold. Samples were determined as testing negative (NEG) for *Bd* DNA if the first qPCR well reaction was negative. Samples were positive (POS) for *Bd* DNA if two of the six replicate qPCR reaction wells were positive. Samples were considered equivalent (EQ) for *Bd* DNA (neither positive nor negative) if only one of the six replicate qPCR reaction wells were positive.

| **Order** | **Identification** | **Site** | **Month** | **No. batches screened**  **(No. of indiv.)** | **No. *Bd*+ batches**  **(No. of indiv.)** | ***Bd* DNA detection result** |
| --- | --- | --- | --- | --- | --- | --- |
| Amphipoda | Amphipoda*^a^*  n = 23 batches; 153 individuals | 2 | Sep | 1 (9) | 0 (0) | NEG |
|  |  |  | Oct | 3 (26) | 0 (0) | NEG |
|  |  |  | Nov | 2 (13) | 0 (0) | NEG |
|  |  |  | Dec | 1 (7) | 0 (0) | NEG |
|  |  | 3 | Oct | 2 (13) | 0 (0) | NEG |
|  |  |  | Nov | 5 (30) | 0 (0) | NEG |
|  |  |  | Dec | 7 (40) | 0 (0) | NEG |
|  |  |  | Jan | 2 (15) | 0 (0) | NEG |
| **Diptera** | **Chironomidae (Adult)**  n = 13 batches; 89 individuals | **1** | **Sep** | **9 (49)** | **1 (3)** | **POS** |
|  |  | 2 | Sep | 1 (10) | 0 (0) | NEG |
|  |  |  | Oct | 3 (30) | 0 (0) | NEG |
|  | Chironomidae (Adult) with parasitic Hydrachnidea*^b^*  n = 1 batch; 5 individuals | 1 | Sep | 1 (5) | 0 (0) | NEG |
|  | Chironomidae (Pupa)  n = 2 batches; 8 individuals | 3 | Oct | 2 (8) | 0 (0) | NEG |
|  | Chironomidae (Larva)  n = 11 batches; 73 individuals | 2 | Sep | 1 (7) | 0 (0) | NEG |
|  |  |  | Dec | 2 (15) | 0 (0) | NEG |
|  |  | 3 | Oct | 1 (3) | 0 (0) | NEG |
|  |  |  | Nov | 4 (30) | 0 (0) | NEG |
|  |  |  | Dec | 1 (7) | 0 (0) | NEG |
|  |  |  | Jan | 2 (11) | 0 (0) | NEG |
|  | Miscellaneous Brachycera (Adult)  n = 3 batches; 30 individuals | 1 | Sep | 3 (30) | 0 (0) | NEG |
|  | Dolichopodidae (Adult)  n = 4 batches; 33 individuals | 1 | Sep | 3 (30) | 0 (0) | NEG |
|  |  | 3 | Jan | 1 (3) | 0 (0) | NEG |
| Anomopoda | Daphnia  n = 3 batches; 30 individuals | 2 | Oct | 3 (30) | 0 (0) | NEG |
| Odonata | Zygoptera (Larva)*^c^*  n = 8 batches; 56 individuals | 1 | Sep | 1 (10) | 0 (0) | NEG |
|  |  | 2 | Oct | 1 (4) | 0 (0) | NEG |
|  |  |  | Dec | 1 (10) | 0 (0) | NEG |
|  |  |  | Jan | 3 (21) | 0 (0) | NEG |
|  |  | 3 | Dec | 2 (11) | 0 (0) | NEG |
|  | Anisoptera (Larva)*^d^*  n = 5 batches; 18 individuals | 1 | Sep | 3 (10) | 0 (0) | NEG |
|  |  | 2 | Dec | 1 (5) | 0 (0) | NEG |
|  |  |  | Jan | 1 (3) | 0 (0) | NEG |
| **Order** | **Identification** | **Site** | **Month** | **No. batches screened**  **(No. of indiv.)** | **No. *Bd*+ batches**  **(No. of indiv.)** | ***Bd* DNA detection result** |
| **Coleoptera** | Dysticidae (Adult)  n = 5 batches; 41 individuals | 2 | Dec | 3 (30) | 0 (0) | NEG |
|  |  |  | Jan | 1 (7) | 0 (0) | NEG |
|  |  | 3 | Oct | 1 (4) | 0 (0) | NEG |
|  | Dysticidae (Larva)  n = 16 batches; 136 individuals | 1 | Sep | 3 (30) | 0 (0) | NEG |
|  |  | 2 | Oct | 3 (21) | 0 (0) | NEG |
|  |  |  | Nov | 4 (36) | 0 (0) | NEG |
|  |  |  | Dec | 3 (26) | 0 (0) | NEG |
|  |  |  | Jan | 3 (23) | 0 (0) | NEG |
|  | Hydrophilidae (Adult)  n = 7 batches; 43 individuals | 1 | Sep | 3 (24) | 0 (0) | NEG |
|  |  | 2 | Oct | 1 (6) | 0 (0) | NEG |
|  |  |  | Nov | 1 (4) | 0 (0) | NEG |
|  |  | 3 | Nov | 1 (4) | 0 (0) | NEG |
|  |  |  | Dec | 1 (5) | 0 (0) | NEG |
|  | **Hydrophilidae (Larva)**  n = 2 batches; 11 individuals | **3** | **Oct** | **2 (11)** | **1 (5);**  **1 (6)** | **POS;**  **EQ** |
| **Hemiptera** | Notonectidae  n = 14 batches; 117 individuals | 1 | Sep | 1 (4) | 0 (0) | NEG |
|  |  | 2 | Nov | 3 (30) | 0 (0) | NEG |
|  |  |  | Dec | 1 (8) | 0 (0) | NEG |
|  |  |  | Jan | 3 (26) | 0 (0) | NEG |
|  |  | 3 | Nov | 3 (30) | 0 (0) | NEG |
|  |  |  | Dec | 2 (15) | 0 (0) | NEG |
|  |  |  | Jan | 1 (4) | 0 (0) | NEG |
|  | Notonectidae with parasitic Hydrachnidea*^b^*  n = 1 batch; 3 individuals | 2 | Jan | 1 (3) | 0 (0) | NEG |
|  | **Corixidae**  n = 19 batches; 169 individuals | **1** | **Sep** | **5 (37)** | **1 (10)** | **POS** |
|  |  | 2 | Oct | 3 (30) | 0 (0) | NEG |
|  |  |  | Dec | 1 (8) | 0 (0) | NEG |
|  |  |  | Jan | 3 (30) | 0 (0) | NEG |
|  |  | 3 | Oct | 3 (25) | 0 (0) | NEG |
|  |  |  | Nov | 3 (30) | 0 (0) | NEG |
|  |  |  | Jan | 1 (9) | 0 (0) | NEG |

*^a^*Amphipods contained members of the family Ceinidae but were mixed with other amphipods from different families that were difficult to identify, particularly because amphipods had lost coloration after storage in ethanol.

*^b^*DNA was extracted from Hydrachnidea parasites along with their invertebrate host. Each invertebrate host had over 20 parasitic Hydrachnidea individuals, and individual numbers in the table refer to number of host individuals.

*^c^*Damselfly larvae included members from Lestidae and Coenagrionidae families but they were not separated out due to insufficient numbers to represent each family separately.

*^d^*Dragonfly larvae included Aeshnid-like dragonflies (Aeshnidae/Telephlebiidae) and corduliid/libellulid-like dragonflies but they were not separated out due to insufficient numbers to represent each family separately.

Table S2| Disease diagnostic testing, qPCR, results for all samples that we determined to be positive for Bd or equivocal. The results here list the number of wells run for the sample, and each line is the qPCR results for each reaction well. When the first reaction was positive for *Bd* DNA we ran that extraction two more times (in triplicate), then re-extracted the sample again and ran those samples in triplicate. For each sample presented here, we report qPCR results for all 6 of the reaction wells. We considered a sample positive for *Bd* if at least 2 reaction wells were positive for *Bd* DNA. A sample was determined to be equivocal is only 1 reaction well was positive for *Bd* DNA. *Bd* DNA copies is the total number of *Bd* ITS gene copies estimated as the total number of ITS DNA copies within the extraction. The positive qPCR well results are in bold

| **Sample ID** | **Taxonomic Group** | **Life Stage** | **Number of individuals within the extraction** | **Site** | **Month** | **Extraction replicate** | **qPCR Reaction status** | ***Bd* DNA copies** | **Number of positive reactions wells (of 6 analyses)** | **Sample determination** |
| --- | --- | --- | --- | --- | --- | --- | --- | --- | --- | --- |
| 15 | Corixidae | Adult | 10 | Site 1 | Sep | 1 | **Positive** | **317** | 2 | *Bd* Positive |
|  |  |  |  |  |  | 1 | **Positive** | **2300** |  |  |
|  |  |  |  |  |  | 1 | Negative | 0 |  |  |
|  |  |  |  |  |  | 2 | Negative | 0 |  |  |
|  |  |  |  |  |  | 2 | Negative | 0 |  |  |
|  |  |  |  |  |  | 2 | Negative | 0 |  |  |
| 96 | Hydrophilidae | Larvae | 6 | Site 3 | Oct | 1 | **Positive** | **550** | 1 | Equivocal |
|  |  |  |  |  |  | 1 | Negative | 0 |  |  |
|  |  |  |  |  |  | 1 | Negative | 0 |  |  |
|  |  |  |  |  |  | 2 | Negative | 0 |  |  |
|  |  |  |  |  |  | 2 | Negative | 0 |  |  |
|  |  |  |  |  |  | 2 | Negative | 0 |  |  |
| **Sample ID** | **Taxonomic Group** | **Life Stage** | **Number of individuals within the extraction** | **Site** | **Month** | **Extraction replicate** | **qPCR Reaction status** | ***Bd* DNA copies** | **Number of positive reactions wells (of 6 analyses)** | **Sample determination** |
| 97 | Hydrophilidae | Larvae | 5 | Site 3 | Oct | 1 | **Positive** | **67** | 2 | *Bd* Positive |
|  |  |  |  |  |  | 2 | **Positive** | **233** |  |  |
|  |  |  |  |  |  | 1 | Negative | 0 |  |  |
|  |  |  |  |  |  | 1 | Negative | 0 |  |  |
|  |  |  |  |  |  | 2 | Negative | 0 |  |  |
|  |  |  |  |  |  | 2 | Negative | 0 |  |  |
| 2b | Chironomidae | Adult | 3 | Site 1 | Sep | 1 | **Positive** | **1317** | 4 | *Bd* Positive |
|  |  |  |  |  |  | 2 | **Positive** | **2167** |  |  |
|  |  |  |  |  |  | 2 | **Positive** | **2183** |  |  |
|  |  |  |  |  |  | 2 | **Positive** | **2383** |  |  |
|  |  |  |  |  |  | 1 | Negative | 0 |  |  |
|  |  |  |  |  |  | 1 | Negative | 0 |  |  |
